# Impact of preoperative biopsy on tumor spread through air spaces in stage I non-small cell lung cancer: a propensity score-matched study

**DOI:** 10.1186/s12890-022-02090-z

**Published:** 2022-07-30

**Authors:** Yun Ding, Jiuzhen Li, Xin Li, Meilin Xu, Hua Geng, Daqiang Sun

**Affiliations:** 1 Clinical School of Thoracic, Tianjin Medical University, Tianjin, China; 2 Department of Thoracic Surgery, Tianjin Chest Hospital, Tianjin, China; 3 Department of Pathology, Tianjin Chest Hospital, Tianjin, China

**Keywords:** Percutaneous needle biopsy, Bronchoscopic biopsy, Tumor spread through air spaces, Prognosis

## Abstract

**Background:**

Percutaneous needle biopsy (PNB) and bronchoscopic biopsy (BB) are widely used in the preoperative diagnosis of pulmonary nodules, but whether PNB or BB may cause tumor spread through air spaces (STAS) has not been reported.

**Methods:**

433 postoperative patients with pathological stage I non-small cell lung cancer (NSCLC) from January 2015 to December 2018 at our hospital were enrolled and divided into PNB group (n = 40), BB group (n = 48) and non-biopsy group (n = 345). The PNB and BB groups were matched using propensity score matched (PSM) separately from the non-biopsy group, after which the effects of PNB and BB on STAS, recurrence-free survival (RFS) and overall survival (OS) were assessed.

**Results:**

After PSM for 9 confounding factors (gender, age, smoking history, tumor site, scope of surgery, pathology type, stage, maximum tumor diameter and postoperative treatment), 38 cases in the PNB group were successfully matched with 38 cases in the non-biopsy group and 28 cases in the BB group were successfully matched with 28 cases in the non-biopsy group. After PSM, there was no significant difference in the incidence of STAS between the PNB and non-biopsy groups (42.1% vs. 34.2%, *P* > 0.05) and between the BB and non-biopsy groups (42.9% vs. 46.4%, *P* > 0.05). The results after PSM showed no significant effect of both PNB and BB on RFS and OS after radical surgery (*P* > 0.05).

**Conclusion:**

Preoperative biopsy in patients with stage I NSCLC has not been shown to increase the occurrence of STAS, nor postoperative recurrence and death.

## Background

Advances in imaging techniques have led to a significant increase in the detection of lung nodules. When the pathological nature of a lung nodule is difficult to determine by imaging, tissue biopsy is often used to make a definitive diagnosis [1]. However, there is controversy as to whether tissue biopsy before surgery in patients with early-stage lung cancer may cause tumor dissemination and affect patient prognosis. This is because tissue biopsy may cause tumor cells to migrate, allowing them to spread in the normal alveolar space or enter the bloodstream, increasing the risk of tumor recurrence after surgery [2, 3]. Surgery is currently the main treatment for early-stage non-small cell lung cancer (NSCLC), but tumor recurrence remains the main cause of postoperative treatment failure [4]. Therefore, we need to fully understand the risk factors for postoperative recurrence of early-stage NSCLC and further clarify whether preoperative invasive biopsy of early-stage NSCLC can lead to tumor dissemination and affect patient prognosis.

Tumor spread through air spaces (STAS) is a newly recognised invasion in lung cancer. In 2015, the World Health Organization (WHO) defined STAS in its new classification of lung cancer as the spread of micropapillary clusters, solid nests and/or single cancer cells spreading within air spaces beyond the edge of the main tumor [5]. Many studies have now found that STAS affects the prognosis of patients with NSCLC [6, 7]. The 2021 WHO Classification of Thoracic Tumours pointed out that STAS is a histologic feature of prognostic significance [8]. Among them, STAS is an important risk factor for tumor recurrence after surgery for stage I NSCLC [9–11]. However, the existence of STAS has also been questioned by many scholars, who believe that STAS may be an artifact of human manipulation due to spreading caused by knife cuts during specimen processing [12], although some studies have been conducted on the specimen retrieval process and confirmed that STAS is an in vivo phenomenon that exists preoperatively and is not a result of spreading caused by specimen processing [13]. Whether STAS is associated with preoperative invasive manipulation has not been reported. Currently, percutaneous needle biopsy (PNB) and bronchoscopic biopsy (BB) are the most commonly used preoperative biopsy techniques and are widely used in the pathological diagnosis of lung nodules suspected of being lung cancer [14–16]. However, there is debate as to whether preoperative biopsy can lead to the spread of lung cancer cells and affect patient prognosis.

This paper reviews the data from patients with stage I NSCLC who underwent surgery at our center to explore the impact of preoperative biopsy, including PNB and BB, on STAS and tumor recurrence.

## Patients and methods

### Study population

**Fig. 1:**
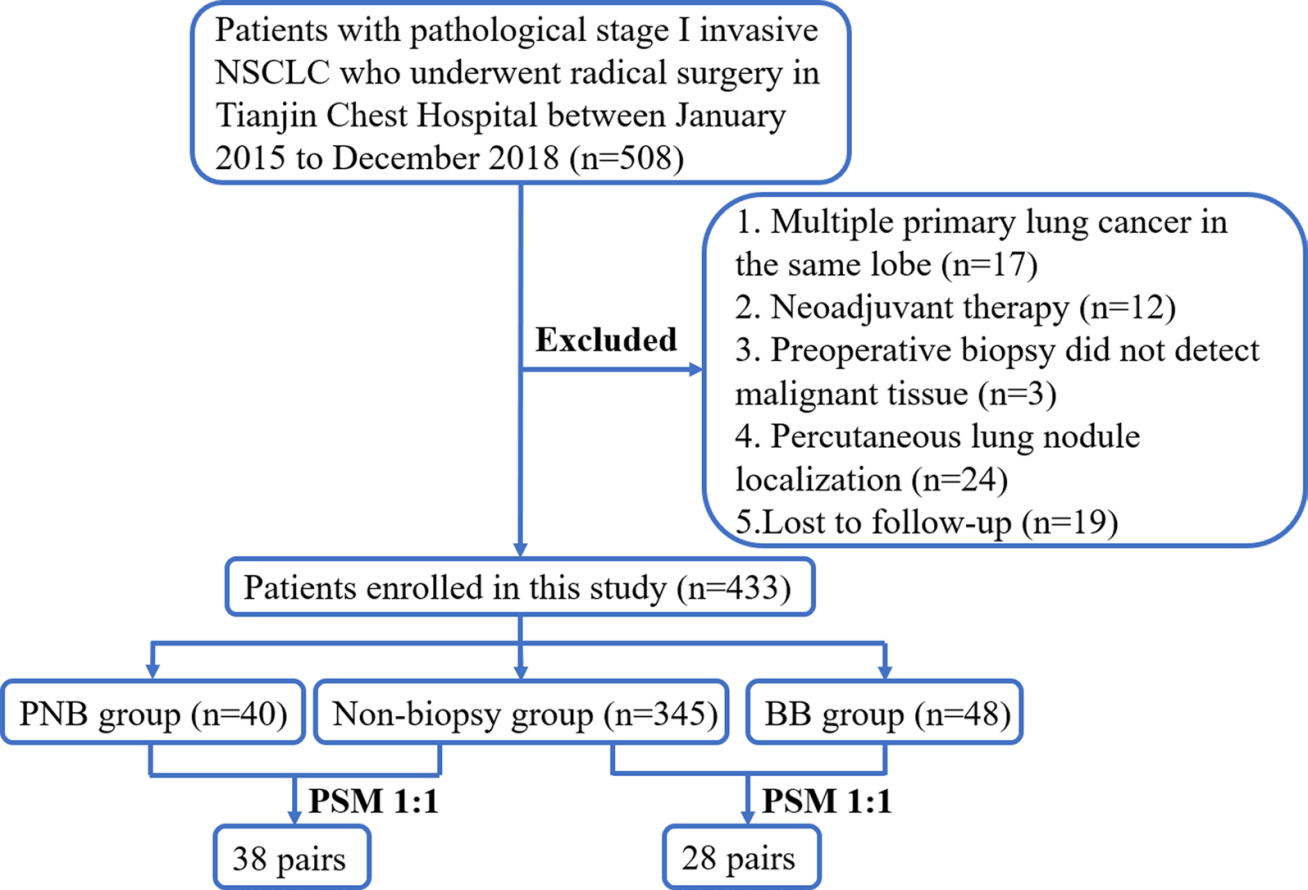
Patients screening flow chart

We reviewed the clinical data of patients who underwent surgical treatment in the department of thoracic surgery of Tianjin Chest Hospital from January 2015 to December 2018 by reviewing our hospital database. Inclusion criteria: underwent complete surgical tumor resection with postoperative pathologically confirmed primary stage I invasive NSCLC. Exclusion criteria: (1) received neoadjuvant therapy before surgery; (2) multiple primary lung cancers in the same lobe; (3) preoperative biopsy did not detect malignant tissue; (4) preoperative percutaneous lung nodule localization; (5) lost to follow-up after surgery. Clinical staging was based on the 8th edition of the American Joint Committee on Cancer TNM staging. Enrolled patients were divided into PNB group, BB group, and non-biopsy group according to preoperative biopsy status. The selection process for patients in this study was displayed in Fig. 1.

### Clinical information

Clinical information included: (1) General information: gender, age, smoking history, tumor site, scope of surgery and postoperative treatment. A positive smoking history was defined as continuous or cumulative smoking for more than 6 months. (2) Preoperative biopsy: whether PNB or BB was performed. All PNB were done under computed tomography (CT) guidance. Notably, broncho alveolar lavage alone was not considered as BB. (3) Postoperative pathology: pathological type, pathological stage, maximum tumor diameter and STAS status.

### Preoperative biopsy technique

PNB was performed under CT guidance. Depending on the location of the tumor under CT, a separate needle tract was selected and punctured. All PNB specimens were obtained by the percutaneous approach using a co-axial needle (18-gauge trocar).

BB was performed under local or general anesthesia using an electronic bronchoscope. The location of the lesion was determined on the basis of bronchoscopic changes in bronchial morphology and mucosa under bronchoscope combined with imaging. Disposable biopsy forceps were used to clamp and collect tumor samples for patients with visible lesions under bronchoscope. Biopsy was performed under endobronchial ultrasound guidance for some patients whose lesions could not be seen directly under bronchoscope.

### Propensity score matching (PSM)

To reduce the effect of possible selective bias, patients in the PNB and BB groups, were matched with those in non-biopsy group for a 1:1 PSM with a caliper value of 0.02, respectively. Matching factors included gender, age, smoking history, tumor site, scope of surgery, pathological type, pathological stage, maximum tumor diameter, and postoperative treatment.

### Evaluation of STAS

**Fig. 2:**
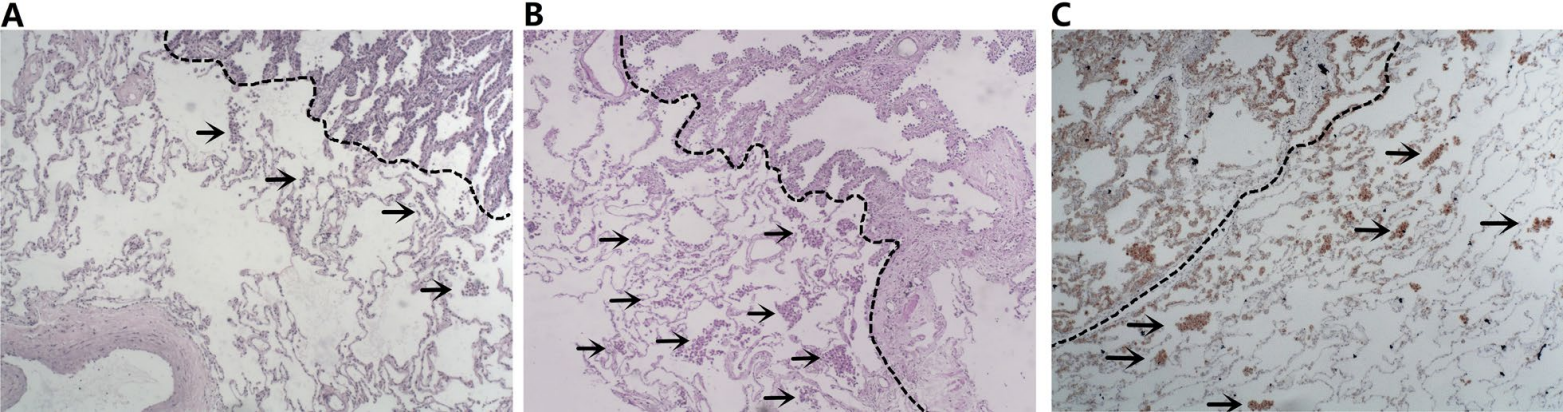
Evaluation of STAS. **A**, **B** Diagnosis of STAS by hematoxylin & eosin staining. STAS (arrowheads) was located within the normal alveolar lumen, outside the margin (black dashed line) of the main tumor. Further, STAS ≤ 3 alveolae away from the main tumor were classified as limited (**A**), STAS > 3 alveolae away from the main tumor were classified as limited extensive (**B**). **C** STAS was identified by immunohistochemistry. STAS (arrowheads, Napsin A positive) was located within the normal alveolar lumen, outside the margin (black dashed line) of the main tumor. STAS: tumor spread through air spaces

Hematoxylin and eosin (HE) sections of surgical specimens from all enrolled patients were retrospectively retrieved and reviewed by the pathologists. The pathologists were not aware of the patients' preoperative biopsies and postoperative prognosis. Referring to the 2015 WHO classification criteria for lung cancer and previous studies [5, 17], we defined STAS positivity as the presence of single tumor cells or clusters of tumor cells in the alveolar lumen at least one alveolar septum away from the main tumor in the section (Fig. 2A, B). Exclusion criteria for STAS [18]: (1) mechanically separated tumor floaters, which were rough clusters of tumor cells randomly distributed or located at the edge of the section; (2) bands of tumor cells detached from the alveolar wall or interstitium due to poor preservation. In cases where it is difficult to differentiate from other tumor-associated cells (e.g., tumor-associated macrophages, dendritic cells, lymphocytes, etc.), the results of immunohistochemistry were referred to determine STAS (Fig. 2C). In addition, referring to the previous study by Warth et al. [19], we classified STAS according to the distance of STAS from the main tumor: limited STAS (≤ 3 alveolae away from the main tumor mass, Fig. 2A) and extensive STAS (> 3 alveolae away from the main tumor mass, Fig. 2B).

### Follow-up visits

Patients were followed up by postoperative visit records review as well as by telephone. Overall survival (OS) was defined as the interval between the date of surgery and the date of death or last follow-up. Recurrence-free survival (RFS) was defined as the interval between the date of surgery and the date of the initial diagnosis of recurrence or the date of the last follow-up. Recurrence included malignant pleural effusion, local recurrence and other distant metastasis.

### Statistical analysis

SPSS 25.0 statistical software was applied for PSM and analysis. Measurement data were expressed as mean ± standard deviation (SD), and t-test was used for comparison between groups. Count data were expressed as percentages (%), and the χ^2^ test or Fisher exact test was used for comparison between groups. The Kaplan–Meier method was used to calculate survival rates and plot survival curves, and the Log-rank test was used for survival analysis. *P*-value < 0.05 was considered a statistically significant difference.

## Results

### Clinical characteristics and STAS of the patients before PSM

Of the total 433 patients, there were 40 in the PNB group, 48 in the BB group, and 345 in the non-biopsy group, and their clinical characteristics are shown in Table 1. Before PSM, the STAS positivity rate was 40.0% (16/40) in the PNB group, 43.8% (21/48) in the BB group, and 40.3% (139/345) in the non-biopsy group. There was no significant difference in STAS positivity rate and STAS degree in the PNB and BB groups compared with the non-biopsy group (*P* > 0.05). However, the proportion of patients with postoperative adjuvant therapy was significantly higher in the PNB group than in the non-biopsy group (*P* < 0.05); the proportion of males, smoking history, central lung cancer and non-adenocarcinoma was significantly higher in the BB group than in the non-biopsy group (*P* < 0.05).

**Table 1:** Clinical characteristics of patients with stage I NSCLC before PSM

Variable	PNB (n = 40)	BB (n = 48)	Non-biopsy (n = 345)	*P*-value*	*P*-value^#^
*Gender*					
Female	20(50.0)	10(20.8)	170(49.3)	0.931	< 0.001
Male	20(50.0)	38(79.2)	175(50.7)		
Age	62.88 ± 8.890	63.52 ± 8.119	62.14 ± 8.052	0.589	0.267
*Smoking history*					
No	27(67.5)	14(29.2)	204(59.1)	0.306	< 0.001
Yes	13(32.5)	34(70.8)	141(40.9)		
*Tumor location*					
Peripheral	37(92.5)	9(18.8)	319(92.5)	1.000^$^	< 0.001
Central	3(7.5)	39(81.3)	26(7.5)		
*Scope of surgery*					
Lobectomy	39(97.5)	47(97.9)	333(96.5)	1.000^$^	1.000^$^
Sublobar resection	1(2.5)	1(2.1)	12(3.5)		
*Pathological type*					
Adenocarcinoma	33(82.5)	8(16.7)	295(85.5)	0.173^$^	< 0.001^$^
Squamous carcinoma	5(12.5)	37(77.1)	46(13.3)		
Other	2(5.0)	3(6.3)	4(1.2)		
*Staging*					
Stage Ia	18(57.1)	30(62.5)	197(57.1)	0.145	0.478
Stage Ib	22(42.9)	18(37.5)	148(42.9)		
Maximum tumor diameter	2.26 ± 0.90	2.34 ± 1.18	2.16 ± 0.80	0.434	0.160
*Postoperative adjuvant therapy*					
No	20(50.0)	30(62.5)	254(73.6)	0.002	0.107
Yes	20(50.0)	18(37.5)	91(26.4)		
STAS					
No	24(60.0)	27(56.2)	206(59.7)	0.972	0.648
Yes	16(40.0)	21(43.8)	139(40.3)		
*STAS degree*					
None	24(60.0)	27(56.2)	206(59.7)	0.191^$^	0.324^$^
Limited	0(0.0)	1(2.1)	24(7.0)		
Extensive	16(40.0)	20(41.7)	115(33.3)		

*NSCLC* non-small cell lung cancer, *PSM* propensity score matching, *PNB* percutaneous needle biopsy, *BB* bronchoscopic biopsy, *STAS* tumor spread through air spaces

*P-value: PNB group versus non-biopsy group

^#^*P*-value 2: BB group versus non-biopsy group

^$^Using Fisher’s exact test

### Clinical characteristics of patients after PSM

As shown in Table 2, 38 patients in the PNB group were matched with 38 patients in the non-biopsy group after PSM, and there was no significant difference in the clinical characteristics of patients between the two groups (*P* > 0.05). Similarly, 28 patients in the BB group were matched with 28 patients in the non-biopsy group after PSM, and again there was no significant difference in the clinical characteristics of patients between the two groups (*P* > 0.05). The sample sizes after PSM met the minimum sample size estimated with PASS software (version 15, two-sided, α err prob = 0.05, power = 0.7).

**Table 2:** Clinical characteristics and STAS of patients with stage I NSCLC after PSM

Variable	PNB (n = 38)	Non-biopsy (n = 38)	*P*-value	BB (n = 28)	Non-biopsy (n = 28)	*P*-value
*Gender*						
Female	20(52.6)	22(57.9)	0.645	6(21.4)	8(28.6)	0.537
Male	18(47.4)	16(42.1)		22(78.6)	20(71.4)	
Age	62.61 ± 9.00	59.24 ± 7.56	0.081	63.11 ± 8.412	63.07 ± 8.08	0.987
*Smoking history*						
No	25(65.8)	27(71.1)	0.622	9(32.1)	9(32.1)	1.000
Yes	13(34.2)	11(28.9)		19(67.9)	19(67.9)	
*Tumor location*						
Peripheral	35(92.1)	38(100.0)	0.240*	9(32.1)	9(32.1)	1.000
Central	3(7.9)	0(0.0)		19(67.9)	19(67.9)	
*Scope of surgery*						
Lobectomy	37(97.4)	38(100.0)	1.000*	27(96.4)	26(92.9)	1.000*
Sublobar resection	1(2.6)	0(0.0)		1(3.6)	2(7.1)	
*Pathological type*						
Adenocarcinoma	32(84.2)	35(92.1)	0.432	7(25.0)	9(32.1)	0.554^#^
Squamous carcinoma	4(10.5)	1(2.6)		21(75.0)	19(67.9)	
Other	2(5.3)	2(5.3)		0(0.0)	0(0.0)	
*Staging*						
Stage Ia	17(44.7)	15(39.5)	0.642	15(53.6)	13(46.4)	0.593
Stage Ib	21(55.3)	23(60.5)		13(46.4)	15(53.6)	
Maximum tumor diameter	2.26 ± 0.84	1.93 ± 0.71	0.074	2.68 ± 1.04	2.53 ± 0.87	0.571
*Postoperative adjuvant therapy*						
No	20(52.6)	19(50.0)	0.818	14(50.0)	11(39.3)	0.420
Yes	18(47.4)	19(50.0)		14(50.0)	17(60.7)	

*NSCLC* non-small cell lung cancer, *PSM* propensity score matching, *PNB* percutaneous needle biopsy, *BB* bronchoscopic biopsy, *STAS* tumor spread through air spaces

^#^Because the Others in both groups are 0, the 2 × 2 chi-square test was performed after deleting this item

*Using Fisher’s exact test

### Status of STAS after PSM

As shown in Table 3, there was no significant difference in the incidence of STAS between the matched PNB and non-biopsy groups (42.1% vs. 34.2%, *P* > 0.05); neither was there a significant difference in the incidence of STAS between the BB and Non-biopsy groups (42.9% vs. 46.4%, *P* > 0.05) after PSM. Similarly, there was no significant effect of both PNB and BB on the degree of STAS (*P* > 0.05).

**Table 3:** Comparison of STAS in patients with stage I NSCLC after PSM

Variable	PNB (n = 38)	Non-biopsy (n = 38)	*P*-value	BB (n = 28)	Non-biopsy (n = 28)	*P*-value
*STAS*						
No	22(57.9)	25(65.8)	0.479	16(57.1)	15(53.6)	0.788
Yes	16(42.1)	13(34.2)		12(42.9)	13(46.4)	
*STAS degree*						
None	22(57.9)	25(65.8)	0.476*	16(57.1)	15(53.6)	0.788^#^
Limited	0	1(2.6)		0(0.0)	0(0.0)	
Extensive	16(42.1)	12(31.6)		12(42.9)	13(46.4)	

*NSCLC* non-small cell lung cancer, *PSM* propensity score matching, *PNB* percutaneous needle biopsy, *BB* bronchoscopic biopsy, *STAS* tumor spread through air spaces

^#^Because the Limited in both groups were 0, the 2 × 2 chi-square test was performed after deleting this item

*Using Fisher’s exact test

### Prognosis of patients before and after PSM

**Fig. 3:**
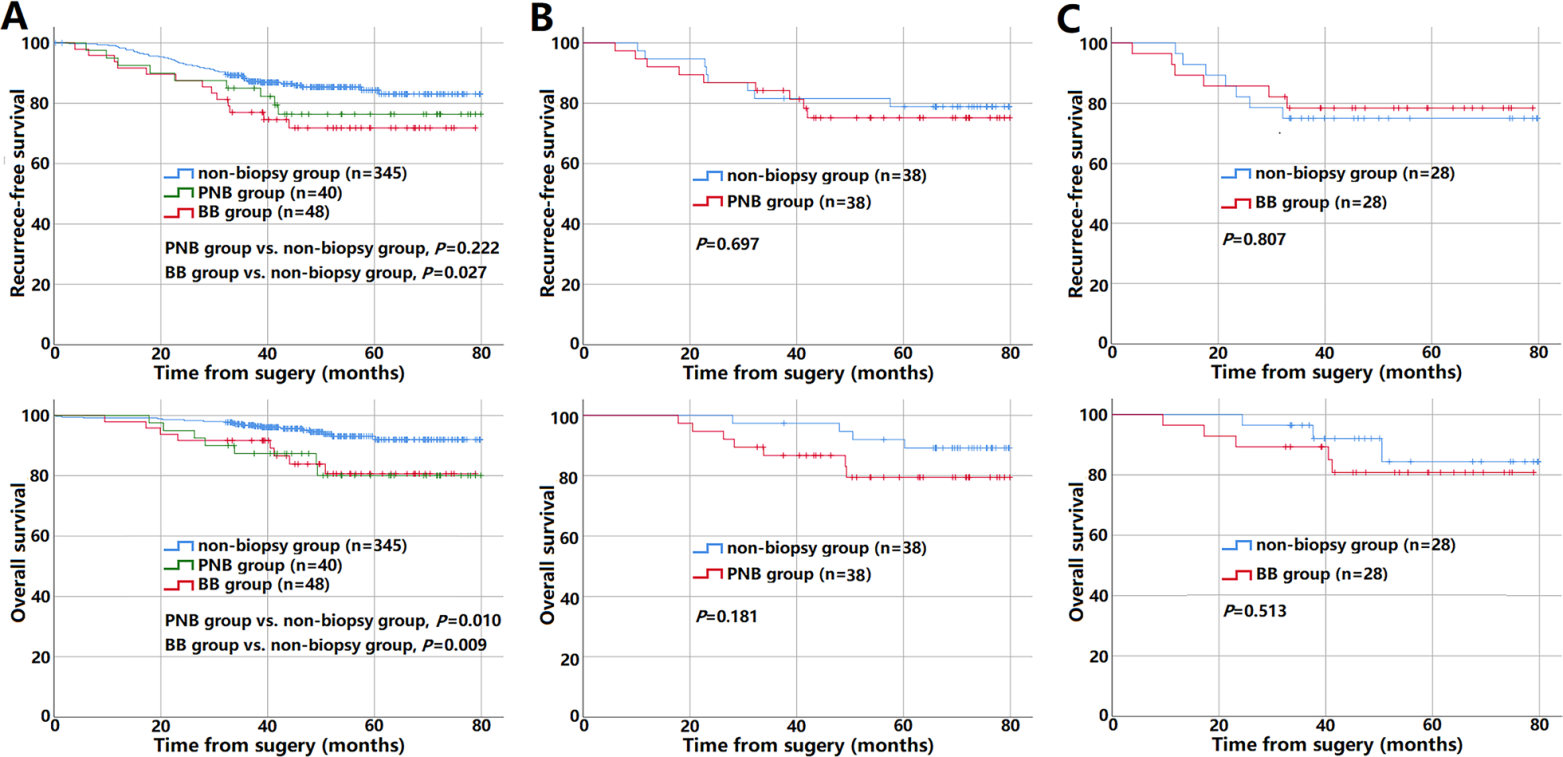
Kaplan–Meier curves of RFS (upper panels) and OS (lower panels) for three groups before PSM (**A**), PNB group versus non-biopsy group after PSM (**B**), and BB group versus non-biopsy group after PSM (**C**)

The median follow-up time of 433 patients was 48.3 months (0.3–80.0 months). Using Kaplan–Meier survival curves to analyze the prognosis of the 433 patients before PSM, there were significant differences in OS between the PNB, BB and non-biopsy groups (PNB group vs. non-biopsy group, *P* = 0.010; BB group vs. non-biopsy group, *P* = 0.009), and RFS between the BB and non-biopsy groups (*P* = 0.027), while there was no significant difference in RFS between PNB and non-biopsy groups (*P* > 0.05), seeing Fig. 3A. Nevertheless, after PSM, there was no significant difference in RFS and OS of PNB group and BB group, respectively, versus the non-biopsy group (*P* > 0.05, Fig. 3B, C).

## Discussion

With the development of imaging techniques, the detection rate of pulmonary nodules has increased, but many of them are benign [20]. Therefore, PNB and BB are commonly used to diagnose pulmonary lesions when they are suspected to be malignant and difficult to differentiate from benign lesions [14–16]. In clinical work, however, some surgeons are concerned that invasive biopsies may lead to the spread of tumor cells, thereby increasing the risk of recurrence after radical resection [2]. They believe that PNB or BB may lead to disruption of the tumor capsule, which may allow tumor cells to migrate and spread in the normal alveolar lumen or enter the bloodstream. Nevertheless, this study using PSM found that preoperative PNB and BB did not significantly increase the rate of STAS in patients with early-stage NSCLC or affect prognosis after radical surgery through PSM analyses.

STAS was a newly recognised mode of invasion of lung cancer cells that has not been widely considered before [21]. It was not until 2015 when WHO formally introduced the concept in the new classification of lung cancer [5]. Many studies have also shown that STAS is significantly associated with worse RFS and OS in lung cancer patients [6, 7]. The reason for this may be that clusters of free-floating tumor cells in the alveolar lumen are deposited in the alveolar wall to form new tumor foci, providing a biological basis for metastasis and recurrence of tumors [22]. However, some scholars have questioned STAS, arguing that it is only the spread of tumor cell clusters caused by knife cutting during specimen processing, and called it "Spread Through a Knife Surface" [12]. Recently, more and more studies have shown that STAS is an in vivo phenomenon rather than an artifact caused by specimen handling procedures [13, 23, 24]. However, could this in vivo phenomenon be another manipulation artifacts, caused by preoperative invasive biopsy? In the present study, neither preoperative PNB nor BB significantly increased the rate of STAS positivity in NSCLC patients either before or after PSM, further suggesting that STAS is an in vivo phenomenon and is not an artifact of preoperative or postoperative manipulation. Of course, it is also possible that tumor cell shedding or dissemination due to invasive manipulation is along the manipulation pathway, i.e., spread is more limited. STAS may not have been observed due to the limitations of pathological sampling [24]. In addition, in this study, the STAS positivity rate was higher in the PNB group than in the non-biopsy group after matching (42.1% vs. 34.2%), but the difference was not statistically significant (*P* > 0.05), probably due to the smaller sample size in both groups after matching. Therefore, further prospective trials with larger sample sizes and comprehensive sampling and serial sectioning of tumor specimens are necessary.

We further analyzed the effect of preoperative biopsy on RFS and OS of patients after radical surgery. While there were significant differences in OS and RFS between the groups before PSM, there were also significant differences in the baseline characteristics of patients between the groups. BB is more suitable for the diagnosis of central lung cancer [25], so the BB group was mostly male patients with squamous carcinoma who smoked, which may lead to different postoperative adjuvant therapies and prognosis in these patients than in the non-biopsy group [26]. Similarly, the selection bias exists for whether to perform PNB on patients preoperatively. Therefore, we performed PSM for the main factors of whether to choose PNB or BB preoperatively, such as the general condition of the patient and the location of the tumor, and for the main factors affecting prognosis, such as the scope of surgery, pathological stage, pathological type, and postoperative adjuvant therapy, to make the patients comparable between groups as much as possible. After PSM, the results showed that preoperative PNB or BB had no significant effect on RFS and OS after radical surgery in NSCLC patients. It is suggested that these diagnostic manipulations are safe and do not significantly affect the prognosis of patients with stage I NSCLC. Several previous studies also support our speculation. Most studies focused on preoperative PNB, as Moon et al. [27] found that preoperative CT-guided PNB may not affect the OS and RFS of patients with radical resection for stage I NSCLC. However, the study by Kashiwabara et al. [28] suggested that PNB may increase the risk of pleural metastases in patients with stage I lung cancer, especially in stage IB. However, in some other studies, no significant effect of preoperative PNB on pleural recurrence has been reported in patients with early-stage lung cancer [29, 30]. Considering that pleural invasion is an important risk factor for ipsilateral pleural recurrence and an indicator of stage IB staging, it is controversial whether patients with pleural invasion are more likely to developed pleural metastasis due to preoperative puncture biopsy. In addition, with the improvement of puncture techniques, the current use of coaxial biopsy needles has also significantly reduced the possibility of needle tract implantation [31]; therefore, preoperative PNB for early-stage lung cancer is still considered safe. In one of the few studies addressing preoperative BB, Hu et al. [32] similarly showed that preoperative BB did not increase the risk of recurrence in patients with resected stage I NSCLC, but the study only compared RFS through stratified analysis without PSM. Our study was refined in this regard and reached similar conclusions, again showing that preoperative BB for early-stage lung cancer is safe without affecting OS and RFS in patients after radical surgery.

The present study also has some limitations. First, since this study was a retrospective study, it was difficult to match the patients exactly despite PSM. Some indicators were not included, such as whether lymph node dissection, visceral pleural invasion, and genetic mutations, because too many indicators might have affected the matching of the cases. Similarly, postoperative adjuvant therapy had an effect on OS, but selection bias for whether to perform adjuvant therapy after surgery and how often to receive it after surgery was difficult to avoid. Moreover, metabolic assessment (e.g., SUVmax) can also be helpful for PSM. However, this retrospective study recruited patients with stage I lung cancer, many of whom did not have metabolic assessment before surgery, so that we did not have sufficient data to include and analyze the metabolic assessment. Second, this was a single-center study with a small number of patients and an even more limited number of patients included after PSM. Therefore, a multicenter study that includes more patients is necessary to be conducted.

## Conclusion

Overall, preoperative biopsy including PNB or BB was not significantly associated with STAS in stage I NSCLC and did not affect RFS and OS in patients with radical surgery for stage I NSCLC, suggesting that STAS is an in vivo phenomenon that may not result from invasive preoperative manipulation. Therefore, these preoperative diagnostic manipulations can be performed when managing patients with suspected early-stage lung cancer.

## Data Availability

All relevant data are within the manuscript.

## References

[1] Gould MK, Donington J, Lynch WR (2013). Evaluation of individuals with pulmonary nodules: When is it lung cancer? Diagnosis and management of lung cancer, 3rd ed: American College of Chest Physicians evidence-based clinical practice guidelines. Chest.

[2] Shyamala K, Girish HC, Murgod S (2014). Risk of tumor cell seeding through biopsy and aspiration cytology. J Int Soc Prev Commun Dent.

[3] Kim YD, Lee BY, Min KO, Kim CK, Moon SW (2014). Intrapulmonary recurrence after computed tomography-guided percutaneous needle biopsy of stage I lung cancer. J Thorac Dis.

[4] Ettinger DS, Wood DE, Aisner DL (2017). Non–Small cell lung cancer, version 5.2017, NCCN clinical practice guidelines in oncology. J Natl Compr Cancer Netw.

[5] Travis WD, Brambilla E, Burke AP, Marx A, Nicholson AG (2015). Introduction to The 2015 world health organization classification of tumors of the lung, pleura, thymus, and heart. J Thorac Oncol.

[6] Toki MI, Harrington K, Syrigos KN (2020). The role of spread through air spaces (STAS) in lung adenocarcinoma prognosis and therapeutic decision making. Lung Cancer.

[7] Shiono S, Endo M, Suzuki K, Hayasaka K, Yanagawa N (2019). Spread through air spaces in lung cancer patients is a risk factor for pulmonary metastasis after surgery. J Thorac Dis.

[8] Nicholson AG, Tsao MS, Beasley MB (2022). The 2021 WHO classification of lung tumors: impact of advances since 2015. J Thorac Oncol.

[9] Yanagawa N, Shiono S, Endo M, Ogata SY (2018). Tumor spread through air spaces is a useful predictor of recurrence and prognosis in stage I lung squamous cell carcinoma, but not in stage II and III. Lung Cancer.

[10] Zombori T, Sejben A, Tiszlavicz L (2020). Architectural grade combined with spread through air spaces (STAS) predicts recurrence and is suitable for stratifying patients who might be eligible for lung sparing surgery for stage I adenocarcinomas. Pathol Oncol Res.

[11] Toyokawa G, Yamada Y, Tagawa T (2018). Significance of spread through air spaces in resected pathological stage I lung adenocarcinoma. Ann Thorac Surg.

[12] Blaauwgeers H, Flieder D, Warth A (2017). A prospective study of loose tissue fragments in non-small cell lung cancer resection specimens: an alternative view to "spread through air spaces". Am J Surg Pathol.

[13] Gross DJ, Hsieh MS, Li Y (2021). Spread through air spaces (STAS) in non-small cell lung carcinoma: evidence supportive of an in vivo phenomenon. Am J Surg Pathol.

[14] Winokur RS, Pua BB, Sullivan BW, Madoff DC (2013). Percutaneous lung biopsy: technique, efficacy, and complications. Semin Intervent Radiol.

[15] Cardoso LV, Souza Júnior AS (2014). Clinical application of CT and CT-guided percutaneous transthoracic needle biopsy in patients with indeterminate pulmonary nodules. J Bras Pneumol.

[16] Katsis JM, Rickman OB, Maldonado F, Lentz RJ (2020). Bronchoscopic biopsy of peripheral pulmonary lesions in 2020: a review of existing technologies. J Thorac Dis.

[17] Kadota K, Nitadori JI, Sima CS (2015). Tumor spread through air spaces is an important pattern of invasion and impacts the frequency and location of recurrences after limited resection for small stage I Lung adenocarcinomas. J Thorac Oncol.

[18] Aly RG, Rekhtman N, Li X (2019). Spread through air spaces (STAS) is prognostic in atypical carcinoid, large cell neuroendocrine carcinoma, and small cell carcinoma of the lung. J Thorac Oncol.

[19] Warth A, Muley T, Kossakowski CA (2015). Prognostic impact of intra-alveolar tumor spread in pulmonary adenocarcinoma. Am J Surg Pathol.

[20] Shen H (2018). Low-dose CT for lung cancer screening: opportunities and challenges. Front Med.

[21] Ma K, Zhan C, Wang S, Shi Y, Jiang W, Wang Q (2019). Spread through air spaces (STAS): a new pathologic morphology in lung cancer. Clin Lung Cancer.

[22] Yagi Y, Aly RG, Tabata K (2020). Three-dimensional histologic, immunohistochemical, and multiplex immunofluorescence analyses of dynamic vessel co-option of spread through air spaces in lung adenocarcinoma. J Thorac Oncol.

[23] Metovic J, Falco EC, Vissio E (2021). Gross specimen handling procedures do not impact the occurrence of spread through air spaces (STAS) in lung cancer. Am J Surg Pathol.

[24] Mino-Kenudson M (2020). Significance of tumor spread through air spaces (STAS) in lung cancer from the pathologist perspective. Transl Lung Cancer Res.

[25] DiBardino DM, Vachani A, Yarmus L (2020). Evaluating the efficacy of bronchoscopy for the diagnosis of early stage lung cancer. J Thorac Dis.

[26] Wang Z, Li M, Huang Y (2018). Clinical and radiological characteristics of central pulmonary adenocarcinoma: a comparison with central squamous cell carcinoma and small cell lung cancer and the impact on treatment response. Onco Targets Ther.

[27] Moon SM, Lee DG, Hwang NY (2017). Ipsilateral pleural recurrence after diagnostic transthoracic needle biopsy in pathological stage I lung cancer patients who underwent curative resection. Lung Cancer.

[28] Kashiwabara K, Semba H, Fujii S, Tsumura S (2016). Preoperative percutaneous transthoracic needle biopsy increased the risk of pleural recurrence in pathological stage I lung cancer patients with sub-pleural pure solid nodules. Cancer Invest.

[29] Ahn SY, Yoon SH, Yang BR, Kim YT, Park CM, Goo JM (2019). Risk of pleural recurrence after percutaneous transthoracic needle biopsy in stage I non-small-cell lung cancer. Eur Radiol.

[30] Matsuoka T, Sonobe M, Date H (2015). Intraoperative fine-needle aspiration biopsy (FNA) for lung cancer: diagnostic value and risk of pleural dissemination. Surg Today.

[31] Yao X, Gomes MM, Tsao MS, Allen CJ, Geddie W, Sekhon H (2012). Fine-needle aspiration biopsy versus core-needle biopsy in diagnosing lung cancer: a systematic review. Curr Oncol.

[32] Hu C, Jiang J, Li Y (2018). Recurrence risk after preoperative biopsy in patients with resected early-stage non-small-cell lung cancer: a retrospective study. Cancer Manag Res.

